# Designing Structural Electrochemical Energy Storage Systems: A Perspective on the Role of Device Chemistry

**DOI:** 10.3389/fchem.2021.810781

**Published:** 2022-01-03

**Authors:** Adriana M. Navarro-Suárez, Milo S. P. Shaffer

**Affiliations:** ^1^ Department of Chemistry, Imperial College London, Molecular Sciences Research Hub, London, United Kingdom; ^2^ Department of Materials, Imperial College London, London, United Kingdom

**Keywords:** structural energy storage, carbon fibers, structural batteries, structural supercapacitors, multifunctional materials, battery chemistry

## Abstract

Structural energy storage devices (SESDs), designed to simultaneously store electrical energy and withstand mechanical loads, offer great potential to reduce the overall system weight in applications such as automotive, aircraft, spacecraft, marine and sports equipment. The greatest improvements will come from systems that implement true multifunctional materials as fully as possible. The realization of electrochemical SESDs therefore requires the identification and development of suitable multifunctional structural electrodes, separators, and electrolytes. Different strategies are available depending on the class of electrochemical energy storage device and the specific chemistries selected. Here, we review existing attempts to build SESDs around carbon fiber (CF) composite electrodes, including the use of both organic and inorganic compounds to increase electrochemical performance. We consider some of the key challenges and discuss the implications for the selection of device chemistries.

## Introduction

Structural energy storage devices (SESDs), or “Structural Power” systems store electrical energy while carrying mechanical loads and have the potential to reduce vehicle weight and ease future electrification across various transport modes (Asp et al., 2019). Two broad approaches have been studied: multifunctional structures and multifunctional materials. The first combines conventional materials by embedding thin-film batteries within composite laminates or sandwich panels. Whilst there can be some synergies and particularly space saving, the structural and energy storage functions generally remain decoupled; *i.e.* one material bears loads, another stores energy electrochemically (Pereira et al., 2009; Thomas et al., 2013). The second approach formulates multifunctional materials that simultaneously and synergistically provide structural and electrochemical energy storage functions (Asp and Greenhalgh, 2014; Danzi et al., 2021). Both approaches have their advantages and challenges, the former offers modest savings under low mechanical loads but suffers from issues such as delamination at the device/composite interface and limited scope for synergy. The latter can potentially offer significantly greater savings in system level mass and volume but the material design is more complicated since the mechanical and electrical demands are often in conflict (Asp et al., 2015).

**FIGURE 1:**
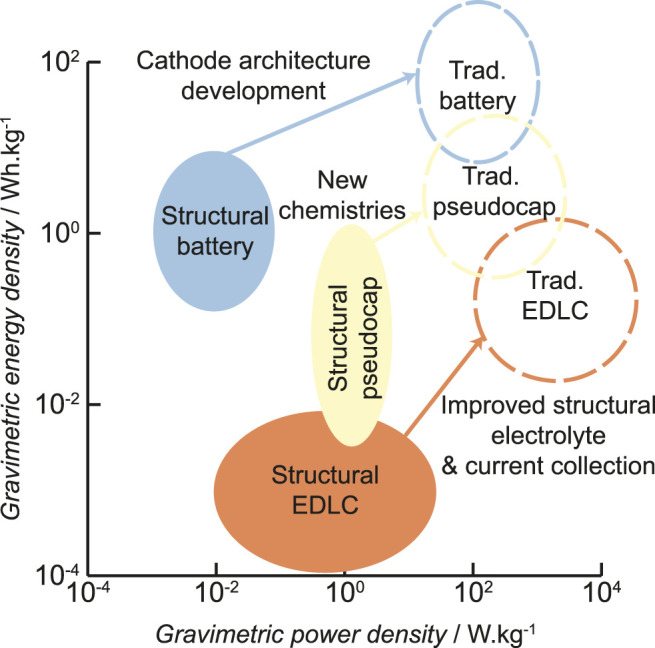
Ragone plot of various SESDs with suggested developments to reach traditional electrochemical energy storage devices.

As discussed further below, SESDs based on fibrous composites are particularly promising. Extensive efforts have been made to identify and address the scientific challenges associated with the underlying multifunctional materials required, including structural electrodes (Liu et al., 2009; Kjell et al., 2011; Shirshova et al., 2013a), electrolytes (Snyder et al., 2007; Ihrner et al., 2017), and separators (Acauan et al., 2019; Patel et al., 2020a). Most of this research has targeted either electrical double-layer capacitors (EDLCs) or lithium-ion batteries (LIBs), leaving aside other chemistries. This focus has been driven by the cycle life/stability of EDLCs and the energy density of LIBs, but potentially misses important opportunities associated with other device chemistries and architectures, covering a wide range of energy and power densities. Novel energy storage concepts incorporating new materials and chemical processes may offer routes to circumvent some key obstacles to existing SESDs (Figure 1) and enable faster implementation in certain applications.

Wider reviews of SESDs are available, particularly describing manufacturing and engineering issues (Asp and Greenhalgh, 2014; Ferreira et al., 2016; Deka et al., 2017a; Danzi et al., 2021; Galos et al., 2021). This perspective examines the prospects for current and future device chemistries, as they may be implemented in SESDs based on fiber composites. The *Current Status Section* summarizes the requirements and current state-of-the-art in SESD chemistry, while the *Future Needs and Prospects Section* considers the selection and possible impact of alternative technologies on device performance.

## Current Status

**FIGURE 2:**
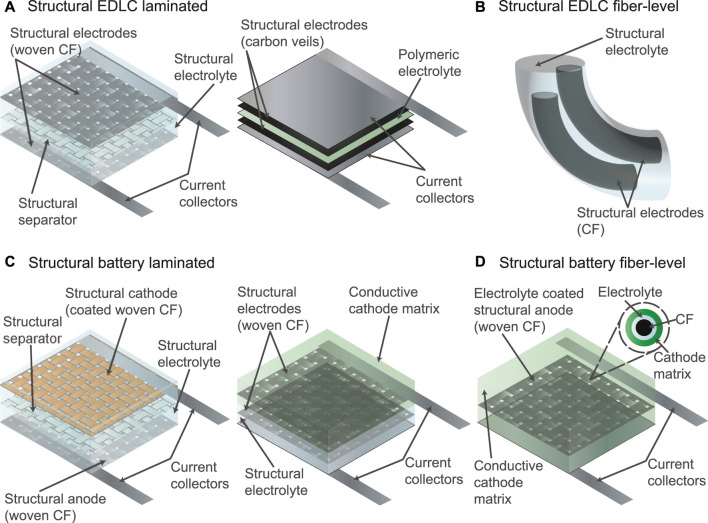
Scheme of **(A)** structural EDLC laminates, **(B)** a structural EDLC fiber-level, **(C)** structural battery laminates, and **(D)** a structural battery fiber-level.

Carbon-based materials, particularly CFs and carbon nanomaterials, are extensively used in SESDs for their electrochemical and structural performance, and low densities. Fiber composites are widely explored as the laminated architecture is common to both electrochemical and structural systems. CFs may be electrochemically active themselves, or act as framework and current collector for a multifunctional matrix packed around them. EDLCs are particularly attractive, since the energy storage process is entirely physical, depending only on the interface between electrode and electrolyte (Figures 2A,B) (Li et al., 2010; Qian et al., 2013a; Shirshova et al., 2013a; Qian et al., 2013b; Javaid et al., 2014; Shirshova et al., 2014; Westover et al., 2014; Greenhalgh et al., 2015; Javaid et al., 2016; Senokos et al., 2016; Kwon et al., 2017; Senokos et al., 2017; Shen and Zhou, 2017; Xu and Zhang, 2017; Li et al., 2018a; Chen et al., 2018; Javaid et al., 2018; Javaid and Irfan, 2018; Muralidharan et al., 2018; Senokos et al., 2018; Aderyani et al., 2019; Flouda et al., 2019a; Chen et al., 2019; Patel et al., 2019; Reece et al., 2019; Patel et al., 2020b; Rana et al., 2020; Reece et al., 2020; Sun et al., 2020; Sánchez-Romate et al., 2021; Subhani et al., 2021; Xu et al., 2021). The central advantage for SESDs, is that there is little or no change in volume, and no (re)dissolution of material, associated with the electrochemical process, minimizing stresses, and simplifying the structural design, whilst ensuring an excellent cycle life. These systems offer modest energy density, and are generally used for their power density in energy management applications, although the long lifetime, reliability, and safety means that they may be used in backup power contexts.

Energy density in EDLCs is broadly proportional to specific surface area, with the caveat that gas adsorption measurements may not reflect the electrochemically accessible regions. In general, the porosity, associated with the necessary high surface areas and electrolyte access, negatively affects mechanical properties, driving many of the efforts to exploit nanomaterials where the perfection of nanostructure may compensate for other performance losses. Whilst many promising systems have been developed, power density is also limited by the challenges of developing an effective structural electrolyte. Ionic conductivity of structural electrolyte “matrices” is usually inversely related to the mechanical properties needed to transfer load (Shirshova et al., 2013b; Ihrner et al., 2017; Schneider et al., 2019). This low ionic conductivity may dominate the equivalent series resistance of the whole device. In addition, whilst structural CFs have reasonable electrical conductivity, in larger devices/components, they may not be an adequate current collector without the addition of additional components. This constraint is more severe in EDLCs than in batteries, as the current densities are expected to be higher.

The balance of power density to energy density can be shifted by incorporating redox active constituents within the stable, high cycle life, porous framework developed for EDLCs. Many researchers have developed pseudocapacitors, by coating carbon electrodes with powders or films that can store charge through surface adsorption/desorption of ions, redox reactions with the electrolyte, or doping/undoping of the electrode materials. Modifications to improve pseudocapacitive behavior have included functional groups (Ganguly et al., 2020), conductive polymers (Benson et al., 2013; Liu et al., 2016; Shi et al., 2016; Hudak et al., 2017; Flouda et al., 2019b; Javaid et al., 2021), metal particles (Mapleback et al., 2020), and metal oxides (Deka et al., 2016; Liu et al., 2016; Deka et al., 2017b; Choi et al., 2017; Deka et al., 2019; Sha et al., 2021).

LIBs exhibit relatively high energy density (∼240 Wh kg^−1^ and 640 Wh L^−1^), long cycle life, relatively high safety, and have a continuously decreasing cost (Armand et al., 2020). The general charge storage mechanism consists of Li ions being stored in, and released from a solid lattice, shuttling back and forth between the anode and cathode. Most of the SESD work in this area has focused on structural anodes, identifying polyacrylonitrile-based CFs with the optimal microstructure to promote good Li-ion intercalation (Wong et al., 2007; Ekstedt et al., 2010; Kjell et al., 2011; Jacques et al., 2012; Jacques et al., 2013; Kjell et al., 2013; Jacques et al., 2014; Hagberg et al., 2016; Feng et al., 2017; Yu et al., 2017; Fredi et al., 2018; Johannisson et al., 2018; Park et al., 2018; Xu et al., 2018; Moyer et al., 2020a; Thakur and Dong, 2020; Xu et al., 2020; Asp et al., 2021). The advantage is that the primary structural fibers act as the energy storage electrode, not simply a current collector; the volume changes may be small enough to be accommodated within the composite, with an appropriate structural electrolyte, and even open up opportunities for high performance actuation (Ferreira et al., 2016). To improve the energy density of the devices, surface modifications with SnO_2_^81^ and MoS_2_-containing anodes have been tried, exhibiting high capacities and excellent cycling stability due to the conversion processes occurring in the SnO_2_ and MoS_2_ anodes.

To date only a limited number of studies on structural cathodes have been published, generally packing the cathode powder into the matrix either around each fiber (Hagberg et al., 2018) or within a separate structural cathode ply (Figures 2C,D) (Galos et al., 2020). The fiber architecture minimizes ionic resistances but increases the risk of shorts; the laminate architecture simplifies fabrication and current collection. Examples of structural LiB cathodes (LiCoO_2_) (Pereira et al., 2007; Liu et al., 2009; Roberts and Aglietti, 2010; Javaid and Ali, 2018), lithium iron phosphate (LiFePO_4_) (Snyder et al., 2006; Ekstedt et al., 2010; Carlson, 2013; Hagberg et al., 2018; Bouton et al., 2019; Moyer et al., 2020b), and lithium nickel manganese cobalt oxide (NMC) (Ladpli et al., 2019). LiCoO_2_ electrodes are a popular choice for use with mobile devices such as smartphones, laptops, and digital cameras (Liu et al., 2015). However, using cobalt raises issues with geo-politics and scarcity, particularly with the projection of at least a tenfold production increase of EVs in the next decade (Li et al., 2020). LiFePO_4_ and NMC are two of the main cobalt-free and low-cobalt battery chemistries that are on the market. LiFePO_4_ batteries benefit from flat voltage profile, low material cost, and abundant material supply, making them ideal for electric transportation. However, they have lower voltage and lower energy density than LiCoO_2_ or NMC (Zhang, 2011). Moreover, they suffer from high electrical resistivity presenting a challenge to develop high cathodic mass fraction for mass efficiency in structural device configurations (Snyder et al., 2015). In NMC, nickel provides the cell with high-specific energy, thus the prevailing trend is to increase the nickel content. However, high-Ni cathode materials generally suffer from lattice and surface instabilities that diminish the longevity of the battery (Lee et al., 2021).

## Future Needs and Prospects

The choice of SESD type and chemistry must be related to the energy/power density required for the chosen application. Recent work has identified different targets with different balances of properties (Nguyen et al., 2021). Very often, in particular applications, the implementation of SESDs will be associated with other system savings, thus the required performance, and potential benefit, is very context dependent. More accessible gains can be accessed by developing secondary systems (*e.g.* aircraft cabin flooring) with more modest energy and power density demands. Here, the best outcomes may be achieved using alternative chemistries; for example, avoiding the fire risk and encapsulation challenges of LIBs, by using aqueous polymeric pseudocapacitors. More ambitious goals (*e.g.* full aircraft electrification) will require dramatic improvements in specific performance; SESDs may be one of the few approaches that can deliver the high effective energy densities required, because the structural functionality may allow much of the device mass to be discounted (Carlstedt and Asp, 2020).

The quest for high energy density structural EDLCs requires CFs to be modified to improve charge storage. Fiber etching to increase surface area typically reduces mechanical strength in fibers, although modest activation of fiber surfaces has been demonstrated without degradation (Shirshova et al., 2014; Snyder et al., 2015). The addition of carbon-based nanomaterials on the fiber or laminate surface can enhance electrochemical surface areas as well as interfacial properties (Anthony et al., 2018; Senokos et al., 2020). Adding nanostructured carbon throughout the multifunctional electrode matrix maximizes the utilization of the available volume (Qian et al., 2013b); however, it is important not to add excess material, as the effective structural fiber volume fraction is then reduced.

Most of the work on structural pseudocapacitors has coated the structural carbon with conductive polymers or metal oxides; usually resulting in a significant decrease in mechanical performance of the composites, although appropriate nanostructuring of the interface may resolve these issues. Transition metal chalcogenides (VS_2_, CuS, CoE_2_, NiS_2_, NiSe_2_), rare-earth metal sulfides (La_2_S_3_ and Sm_2_S_3_), and layer-structured chalcogenides (MoS_2_ and SnSe) have been previously proposed as attractive electrode materials for flexible devices due their ability to host redox reactions and the tunable gap between the layers that can intercalate ions from the electrolyte (Palchoudhury et al., 2019). MXenes, 2D transition metal carbides and nitrides, have also shown promising results in flexible supercapacitors (Zhao et al., 2015; Yan et al., 2017; Zhang et al., 2017; Couly et al., 2018; Zheng et al., 2019), in both aqueous and ionic liquid electrolytes. Both transition metal (di) chalcogenides and MXenes could be extended to SESDs. To minimize the effect of modulating the primary fiber-matrix interfaces, and maximize the active volume, these species can be incorporated within a multifunctional matrix material, throughout the structural electrode; electrical conductivity can be supplemented by carbon nanomaterials or a continuous carbon aerogel network (Qian et al., 2013b), if required. In this case, the primary CFs may simply act as structural current collector. The contribution of the structural matrix will be limited by the available matrix volume fraction. Increasing the matrix volume fraction will not generally be attractive as the primary structural fiber volume fraction will fall, significantly degrading structural performance.

Most of the studies on structural LIBs used CFs as anodes, but many aspects, including dendrite formation and crack nucleation during the lithiation/delithiation cycles, have yet to be deeply addressed (Danzi et al., 2021). Interfacial adhesion between CFs and the structural electrolyte will be vital for the mechanical and electrochemical properties of SESDs; thus *operando* and modelling studies are needed to analyze the inherent behaviors, particularly over multiple cycles. The problem is even more complex where additional coatings of high-capacity materials are applied, although nanoscale texture can assist load transfer (Qian et al., 2008; Qian et al., 2010; Mikhalchan and Vilatela, 2019). The electrochemical performance of existing CFs has been enhanced by, for example, decorating them with different metal oxides (*e.g.* NiO, SnO_2_, ZnO, ZnCo_2_O_4_) (Han et al., 2019; Han et al., 2020a; Han et al., 2020b; Han et al., 2020c). All these composites exhibited enhanced Li storage performance. However, no mechanical studies were performed; since the electrochemical testing was performed with a conventional, rather than multifunctional electrolyte, their suitability for SESDs is not yet clear. Computational studies have proposed Co_3_O_4_ as structural anode (Hu et al., 2021); experimental testing is yet to be performed. Most of high-capacity anode materials, including Co_3_O_4_ and silicon, exhibit low cycling stability, due to volume expansion during Li insertion/extraction. In general, the main challenge for these high energy density insertion anodes, whether designed at either fiber or laminate level, may remain how to accommodate volume changes during cycling within a structural composite material.

Due to its high theoretical capacity (3860 mAh g^−1^), low density (0.59 g cm^−3^) and negative electrochemical potential (−3.04 V *vs*. SHE), Li metal has many attractions as anode for batteries. However, Li-metal presents particular challenges with safety, due to potential dendrite formation and associated fire risk. In any case, in a SESD context, the pure metal will make little mechanical contribution, both due to its intrinsic properties, and its structure as it dissolves and redeposits. Reactive anodes exploiting silicon, tin, or phosphorus, similarly may have limited value as the large volume changes may be hard to accommodate, and cycle life tends to be low. However, these additional components could be incorporated within a multifunctional matrix approach. Thermal runaway in case of damage by mechanical loads is a concern in all SESD but maybe be mitigated by the intrinsically distributed energy storage, large surface area for cooling, and opportunities for self-limiting delamination (Kalnaus et al., 2021). Nevertheless, where high volumetric and gravimetric energy densities are needed, primary aluminum–air and secondary zinc–air may offer better damage tolerance than Li systems (Hopkins et al., 2020). 3D printing has been proposed as an appropriate fabrication method for structural metal-oxygen batteries as the micro-structures and shapes of electrodes/electrolytes/current collectors/packaging materials can be controlled, improving mechanical and electrochemical performance (Zeng et al., 2020). However, the autophageous nature of pure metal batteries may limit their relevance as SESDs.

Conversion-type cathodes, such as sulfur, and oxygen, typically store 2-3 Li-ions per anion and therefore could potentially exhibit higher theoretical energy densities compared to the current LIBs (*e.g.* Li−O_2_, 3505 Wh kg^−1^; Li−S, 2600 Wh kg^−1^) (Guo et al., 2017). Even so, they suffer from low conductivity (leading to low capacity utilization, poor kinetics, and poor reversibility of conversion reactions) and voltage hysteresis (typically linked to poor electronic resistance of the materials leading to low energy efficiency) (Wu and Yushin, 2017). Traditionally, conductive carbons, conducting polymers, metal organic frameworks and various metal oxides have been used to improve the conductivities of some conversion cathodes and reduce their dissolution during cycling (Guo and Fu, 2018). Conversion-type cathodes may be attractive in SESDs as CFs and CF-supported aerogels (Qian et al., 2013b; Nguyen et al., 2019) could potentially solve the low conductivity and voltage hysteresis issues. To date, there has only been one approach towards structural Li-S batteries (Huang et al., 2020); yet its electrolyte-to-sulfur weight ratio (∼27) was extremely high for practical applications given that an excess of electrolyte (ratio >4) has an adverse impact on the energy density of the battery at the system level. Moreover, the energy density reported is calculated based on the sulfur contained in the electrode, ignoring the sulfur in the catholyte, thus, overestimating the energy density of the device in a practical sense.

Concerns about Li resource shortages have led to an interest in alternative chemistries in battery applications. CFs have been tested as anodes in potassium-ion (Harnden et al., 2021) and sodium-ion batteries (Harnden et al., 2018). Potassium-insertion gives a larger voltage-strain coupling than Na probably due to a slightly higher ionic diffusion coefficient; therefore, potassium-insertion appears more promising for creating actuation multifunctionality. Still, Li-insertion appears most promising for high-performance SESDs, where mass is the underlying driver.

As a more radical alternative to LIB or other high voltage systems, aqueous zinc batteries may be attractive due to the low cost of Zn, its low toxicity, low flammability, high stability and compatibility in aqueous electrolytes, as well as a high theoretical anode gravimetric and volumetric capacity (820 mAh g^−1^, 5851 mAh cm^−3^) (Jia et al., 2020). A CF Zn–MnO_2_ structural composite battery was tested using a gel electrolyte exhibiting reasonably high stiffness and electrochemical performance (Chen et al., 2021). As the energy density was calculated per active material, instead of per device, it is difficult to assess the weight saving benefits of the SESDs, but an embodiment with a multifunctional matrix electrode could prove beneficial. The use of an aqueous electrolyte has obvious attractions for SESDs, reducing fire risk, toxicity, and minimizing encapsulation challenges.

The fabrication of batteries with inorganic cathodes is costly and environmentally damaging, from the extraction of the transition metals in ores with low metal content (Mauger et al., 2019), to the preparation of the active materials. Organic electroactive components can be used in both pseudocapacitors and organic batteries (small molecule or polymer) (Muench et al., 2016; Chen et al., 2020; John et al., 2020) and may offer environmental benefits both in production and recycling. In principle, these organic electrodes are composed of naturally abundant elements and they are less environmentally challenging compared to metal-based batteries. Both n- and p-type storage mechanisms can be implemented in organic electrodes, enabling various cell or electrode configurations (Poizot et al., 2018). The production of CFs themselves is relatively energy intensive; whilst over their lifetime, they may make a positive contribution to reducing emissions, though improved fuel efficiency in application, there is significant interest in developing “greener” CFs from renewable resources. Whilst absolute mechanical performance is relatively low, it is approaching the range needed for automotive applications and there may be opportunities for new types of multifunctional CFs (Mariano et al., 2014; Baker and Rials, 2013).

## Concluding Remarks

Structural energy storage devices are a promising approach to reduce the weight of the battery pack, and hence increase range, in electric transportation. Many advances have been made in CF for structural EDLCs and LIB anodes, although the development of effective structural electrolytes remains challenging. The fiber-matrix interface also needs to be optimized to ensure both load transfer and electrochemical access, as well as accommodating intercalation-induced strains in structural batteries. Electrochemical performance can be enhanced by adding additional electroactive components, either coated at the fiber/lamina surface, or more beneficially incorporated throughout the multifunctional electrode matrix volume. There is significant scope to develop this concept, which is likely to be essential to developing structural cathodes to complement existing structural anodes.

Whilst high energy densities are a key driver, other factors may be more crucial; in some applications, more modest performance can offer significant benefits when the whole system is considered. In SESDs, longevity is particularly important, as the energy storage function is an inherent part of the whole product and cannot easily be replaced. In addition, the distribution of the electrochemical system over a large area, where fastenings and other connections are required, makes encapsulation and air-free fabrication challenging. SESDs may therefore particularly benefit from high cyclability systems, and aqueous, or at least water-tolerant, chemistries. Fire, smoke, and toxicity concerns will vary with application. The distributed character of the energy storage in SESDs tends to mitigate fire risk, and initial trials have indicated benign failures (Kalnaus et al., 2021); however, chemistries may be selected to further reduce risk. In addition, materials researchers are increasingly considering the sustainability and full life cycle analysis of new systems; there is significant scope to implement bio-derived materials, for both carbonaceous and organic redox-active constituents.

Fair performance comparisons in multifunctional applications can be difficult, but a variety of methodologies are emerging (Carlstedt and Asp, 2020; Johannisson et al., 2020; Nguyen et al., 2021). As well as the intrinsic electrochemical performance of different chemistries, it is important to consider device energy densities in existing embodiments and projected to future embodiments that might be compatible with the given storage system. Consideration must be given to the optimal architecture (fiber or laminate level), the need for current collection, and encapsulation, as well as the implications for mechanical integrity and load-carrying capacity. New modelling approaches are needed that combine the relevant thermomechanical-electrochemical processes. SESD composites represent an important and stimulating field of research, that requires interdisciplinary collaborations to accelerate progress towards real world deployment.
